# Dr. William D. Haggard

**Published:** 1901-04

**Authors:** 


Dr. William D. Haggard, of Nashville, Tenn., died at Columbia in that state January 25, 1901, from the effects of an attack of paralysis which occurred on the previous day, aged 74 years. Dr. Haggard was one of the best known physicians in the South, and was a teacher and surgeon of great ability. He leaves a son, Dr. William D. Haggard, Jr., who is rapidly attaining a similar professional eminence.

